# Adapting Effective mHealth Interventions to Improve Uptake and Adherence to HIV Pre-Exposure Prophylaxis Among Thai Young Men Who Have Sex With Men: Protocol for a Randomized Controlled Trial

**DOI:** 10.2196/46435

**Published:** 2023-09-04

**Authors:** Bo Wang, Rena Janamnuaysook, Karen MacDonell, Chokechai Rongkavilit, Elizabeth Schieber, Sylvie Naar, Nittaya Phanuphak

**Affiliations:** 1 Department of Population and Quantitative Health Sciences University of Massachusetts Chan Medical School Worcester, MA United States; 2 Institute of HIV Research and Innovation Center of Excellence in Transgender Health Chulalongkorn University Bangkok Thailand; 3 Center for Translational Behavioral Research Florida State University Tallahassee, FL United States; 4 Department of Pediatrics University of California San Francisco Fresno Branch Campus Fresno, CA United States

**Keywords:** young men who have sex with men, pre-exposure prophylaxis, PrEP, HIV prevention, mHealth, motivational interviewing, motivational text messaging, Thailand

## Abstract

**Background:**

Young men who have sex with men (YMSM) are the fastest-growing HIV-positive population worldwide. Thailand has the highest adult HIV seroprevalence in Asia; over 25% of men having sex with men in Bangkok are HIV positive. Pre-exposure prophylaxis (PrEP) is an efficacious HIV prevention strategy recommended for all at-risk individuals. PrEP is highly effective when taken as prescribed, but PrEP utilization rate has been low, and adherence is often inadequate.

**Objective:**

We propose to develop and pilot a multicomponent, technology-based intervention to promote motivation to begin PrEP (“uptake”) and sustained adherence to PrEP among HIV-negative Thai YMSM. We will adapt an existing 2-session technology-delivered, motivational interviewing–based intervention to focus on PrEP use in YMSM in Thailand. The resulting intervention is called the Motivational Enhancement System for PrEP Uptake and Adherence (MES-PrEP). We will also develop motivational text messaging (MTM) to send two-way motivational messages to promote PrEP use.

**Methods:**

The proposed study includes 3 phases. Phase 1 includes in-depth interviews with HIV-negative Thai YMSM and providers to explore barriers and facilitators of PrEP initiation and adherence, aiming to inform intervention content. Phase 2 consists of adapting and beta-testing MES-PrEP and MTM for functionality and feasibility using a youth advisory board of Thai YMSM. In Phase 3, we will conduct a pilot randomized controlled trial to evaluate the feasibility, acceptability, and preliminary efficacy of MES-PrEP and MTM to increase PrEP uptake and adherence among Thai YMSM. A total of 60 HIV-negative Thai YMSM who have not started PrEP and 60 YMSM who are on PrEP but not adherent to it will be randomized 2:1 to receive MES-PrEP and MTM (n=40) or standard PrEP counseling (n=20). The feasibility and acceptability of the intervention will be assessed through usage patterns and the System Usability Scale. The preliminary impact will be assessed by evaluating the proportion of PrEP initiation and level of adherence to PrEP. Participants will complete the assessments at baseline and at 1-, 3-, and 6-month postintervention. Biomarkers of adherence to PrEP and biomarkers of HIV and sexually transmitted infections will be collected.

**Results:**

Recruitment for this study began in January 2022 for phase 1. Qualitative interviews were completed with 30 YMSM and 5 clinical providers in May 2022. Phase 3, the pilot feasibility and acceptability trial, began in July 2023. Upon project completion, we shall have developed a highly innovative mobile health intervention to support YMSM using PrEP, which will be ready for testing in a larger efficacy trial.

**Conclusions:**

This study addresses a critical problem (ie, high HIV incidence and low PrEP use) among Thai YMSM. We are developing 2 potentially synergistic technology-based, theory-driven interventions aimed at maximizing PrEP use. The proposed project has the potential to make significant contributions to advancing HIV prevention research and implementation science.

**Trial Registration:**

ClinicalTrials.gov NCT05243030; https://clinicaltrials.gov/ct2/show/NCT05243030

**International Registered Report Identifier (IRRID):**

DERR1-10.2196/46435

## Introduction

### Overview

Men who have sex with men (MSM) are 19 times more likely to be HIV positive than the general population worldwide, with an HIV prevalence of 20%-30% in many countries [1-4]. New infection rates are particularly high in young MSM (YMSM), whose developmental period is associated with increased risk-taking [5]. Thailand has the highest adult HIV seroprevalence in Asia (1.1% in 2017) [6]. The rate of new HIV infections among MSM dramatically increased in the past 20 years [7], with an HIV prevalence rate reaching 30% and an incidence rate of 12 per 100 person-years among those aged 15-21 years [4]. Effective interventions for this high-risk population are critical to the success of the UNAIDS’ Fast Track Strategy—ending the AIDS epidemic by 2030 [8].

Pre-exposure prophylaxis (PrEP) is an efficacious HIV prevention strategy. The Centers for Disease Control and Prevention recommends PrEP for all individuals at substantial risk for HIV infection, including HIV-negative MSM [9]. Despite high levels of efficacy when taken as prescribed, PrEP uptake and adherence have been low [10], with high discontinuation rates reported among YMSM [11,12]. The existing literature suggests that YMSM generally lack awareness of PrEP and their uptake motivation and adherence are often inadequate [13-15]. In Thailand, only 9.3% of MSM who were offered PrEP agreed to take it [16]. Commonly cited barriers to initiating or staying on PrEP have included low self-perceived risk, concerns about medication side effects, concerns about maintaining daily oral PrEP regimen, drug use, and HIV stigma [17-19]. Given this evidence of low uptake and high discontinuance rates of PrEP, effective behavior-change interventions are needed to motivate the uptake of and sustain adherence to PrEP among HIV-negative YMSM in Thailand.

Mobile health (mHealth) interventions for high-risk YMSM have the potential to reduce HIV risk behaviors and increase HIV testing rates [20]. These interventions may be particularly well suited for youth because they tend to use computers, cell phones, and texting very frequently [21,22]. Motivational interviewing (MI) is the only evidence-based behavioral intervention that has demonstrated success across the youth HIV prevention and care cascades [23-25]. MI has been linked with improved medication adherence, decreased depression, and decreased risky sexual behaviors in people living with HIV [26]. Interactive text messaging provides ongoing support to maintain motivation for behavior change. Text messaging has a beneficial impact on antiretroviral therapy adherence and provides ongoing behavioral reinforcement for HIV-positive MSM, and the effect is stronger when combined with other interventions [27,28]. Despite the rapid growth of technology in behavioral health, there have been very few technology-based interventions that have specifically targeted HIV-negative YMSM for PrEP uptake and adherence.

### Study Aims

We propose to develop and pilot a multicomponent, technology-based intervention to promote both motivation to begin PrEP (“uptake”) and sustained adherence to PrEP. Intervention development and testing will be guided by the framework of the ADAPT-ITT model [29]. First, we will adapt an existing 2-session technology-delivered, MI-based intervention to focus on PrEP uptake and adherence in HIV-negative YMSM through a systematic, multistep process [30]. The resulting brief technology-based intervention will be called the Motivational Enhancement System for PrEP Uptake and Adherence (MES-PrEP). We shall develop and pilot-test motivational text messaging (MTM) to promote PrEP uptake and adherence. These intervention components will be integrated to enhance the impact of MES-PrEP through MTM. Second, we will conduct a pilot randomized controlled trial (RCT) to evaluate the feasibility, acceptability, and preliminary efficacy of an intervention combining MES-PrEP and MTM to increase PrEP uptake and adherence in Thai YMSM. This study will pave the way for the successful scale-up of PrEP implementation among MSM in Thailand.

### Theoretical Frameworks of Behavior Change

The Information-Motivation-Behavioral Skills (IMB) model and the Socioecological Model will serve as the conceptual frameworks for developing our PrEP mobile intervention. Both theoretical models have been applied to a variety of health promotion topics, including HIV health behaviors [31-33] and PrEP uptake and adherence [34,35]. Although the IMB model includes only individual-level factors, the Socioecological Model captures individual-, structural-, and social-level factors around PrEP use. The proposed intervention is primarily focused on behavior change at the level of the individual and also addresses structural- and social-level factors, such as HIV stigma and depression as well as access to PrEP. According to the IMB model, behavior change results from the joint function of 3 critical elements: accurate information about risk behaviors (eg, risks of not taking PrEP while engaging in unprotected sex), the motivation to change behavior, and the perceived behavioral skills necessary to perform the behavior (eg, self-efficacy). For this study, we will develop intervention content to address each core element of the IMB model to facilitate PrEP adoption and adherence among Thai YMSM.

### Intervention Components

#### MES-PrEP Intervention

MES-PrEP is a 2-session computer-delivered intervention. At the start of each session, the participant chooses a preferred character (avatar) who will deliver the intervention in a way that has high fidelity to the most recent edition of MI (MI-3) [36]. The avatar evokes both importance and confidence (key components of readiness or motivation) with MI strategies, such as identifying the pros of behavior change, providing affirmations to reinforce change talk and boost confidence, as well as identifying strengths and resources. MI strategies used within the motivational enhancement system to promote autonomy, boost self-efficacy, and identify social supports are also designed to address HIV stigma and coping. The intervention manages counterchange talk by having the avatar reflect without judgment and providing statements to emphasize autonomy. Importantly, the interactions between the avatar and the participant are synchronous and not reliant on feedback at the completion of the session. Small amounts of education about PrEP are integrated with motivational elements. The intervention is adjusted based on participants’ ratings of the perceived importance of and confidence in taking PrEP by routing each participant to different content based on these assessments. For example, participants who perceive initiation of PrEP to be of low importance are branched to decisional balance exercises, while participants who perceive this to be very important receive reinforcement for this perception. Youth, regardless of the level of importance and confidence, are also directed to content designed to explore HIV stigma, including exploration of autonomy, self-efficacy, and social support. Youth are given feedback on their PrEP knowledge assessment followed by information about the protective effect that can result from improved PrEP adherence. Each participant will be asked to set a goal (obtain PrEP prescription, maintain optimal adherence, practice steps, or think about it more), identify possible barriers (eg, concerns around stigma, forgetting, and side effects), and form plans for overcoming these barriers. In the second session (1 month later), participants are assigned to new branches based on whether they felt that they met their goal (reinforcement and plan for continued success), partially met the goal (identifying plans for overcoming barriers), or did not meet the goal. Youth may choose to plan for a new goal or continue the same goal.

#### MTM Intervention

Text messages are motivational statements promoting the uptake of and adherence to PrEP. We will use LINE for automated but bidirectional MTM to send and receive text messages to promote PrEP initiation and adherence. The program features automatic prompts and response data capturing, requires no internet, and works in the Thai language. The interface program will allow flexibility in the timing and frequency of MTM; message selections from the message libraries; and automatic recording of the message sent, the message received, and whether the message fails. All MTM activities will be performed by an information technologist at the Institute of HIV Research and Innovation. MTM will be sent daily for 1 month, followed by weekly for an additional 5 months (months 2-6) at a time chosen by the participant to promote autonomy. The message content is individualized based on participant responses to the baseline survey. Those participants who indicate that they are “ready” to initiate PrEP and set the goal to take their medications as prescribed will receive MTM to remind them to take PrEP. Those who indicate that they are less ready to take PrEP can choose from alternatives, including taking on-demand PrEP or just thinking about taking PrEP. MTM content for those who are not ready to take PrEP will be individualized based on the participant’s choice, that is, a daily message encouraging them to work toward their chosen goal. Participants who are not prescribed PrEP receive daily text messages regarding PrEP effectiveness, HIV risk, and getting prescribed PrEP.

#### Standard PrEP Counseling (Control Condition)

All study participants will receive one-on-one, face-to-face counseling focused on sexual and behavioral risk assessment for HIV and sexually transmitted infections as well as risk reduction. For those who are at high risk but not yet ready to start PrEP, the focus will be on risk assessment skills or risk perception, awareness of PrEP or postexposure prophylaxis, as well as facilitators and barriers to accessing PrEP. For those already on PrEP, the focus will be on adherence. Counseling is a standard practice within our partner clinics and will last about 15-20 minutes.

## Methods

### Study Setting

YMSM will be recruited from 2 large clinics in Bangkok, Thailand, to ensure diversity in the respondent population. Thai Red Cross Anonymous Clinic has a large YMSM client population. Over 3500 YMSM (aged 16-25 years) attended this clinic in 2020 [37]. Rainbow Sky Association of Thailand (RSAT) clinic is among the first community-based organizations working to serve key populations in Thailand. RSAT has implemented the Key Population-Led Health Services model since 2015 in a highly populated area in Bangkok to provide HIV-related health services for key populations, including MSM. Annually, approximately 1500 YMSM (aged 16-25 years) have attended the RSAT clinic.

### Study Design

There are 3 phases in the study: phase 1 (formative research), phase 2 (intervention adaptation), and phase 3 (pilot RCT).

#### Phase 1: Formative Research

##### In-Depth Interviews

We will conduct interviews with 30 HIV-negative Thai YMSM (15 PrEP-naïve YMSM and 15 YMSM on PrEP, including 5 with good adherence) to explore factors related to PrEP uptake and adherence. An interview guide will be created by the investigator team that will provide a general structure for the interview. Based on the IMB model, we will explore their “information” (ie, their knowledge of PrEP and HIV transmission risk and their perceived advantages and disadvantages of PrEP), “motivation” (ie, attitudes toward PrEP, perceptions of HIV risk, willingness and interest to use PrEP, reasons for accepting or declining PrEP), and “behavioral skills” (ie, specific behavioral skills relevant to PrEP initiation and adherence, facilitators and barriers to PrEP initiation and adherence, reasons for keeping scheduled appointments or filling PrEP prescription, and reasons for discontinuing or restarting PrEP). We will also explore sources of social support and past successes. Responses will be used to broadly inform intervention content (eg, which modules to include in the intervention framework). The responses will also be used for more specific purposes (eg, response options for barriers and identifying sources of social support). We will also prompt the participants for feedback on intervention content (eg, what they would like to know about PrEP) and delivery (eg, how often they would like to receive text messages to help them take PrEP). Interviews will be audiotaped, transcribed, and translated into English by an independent translator. Interviews will last about 30 minutes.

##### Youth Advisory Board

A youth advisory board (YAB) of 8-10 eligible Thai YMSM will be identified and assembled by Thailand investigators who have strong relationships with medical providers serving YMSM in Bangkok. YAB members will be high-risk YMSM and selected to ensure diversity in age and socioeconomic status. Members will meet quarterly (with additional meetings as needed). The primary role of the YAB will be providing iterative feedback on study materials, recruitment and retention strategies, intervention approach and content, and disseminating study findings. The operational guidelines will follow the best processes for community-participatory research (eg, establishing the YAB’s function and promoting empowerment) [38]. Any inconsistencies in feedback will be resolved during the meeting, or if necessary, via discussions with the Thai-US study team. Detailed notes will be taken during meetings. Study materials will be revised based on YAB feedback.

#### Phase 2: Adapting and Refining MES-PrEP and MTM

##### Adapting Motivational Enhancement System for Adherence to Focus on PrEP Uptake and Adherence (Development of “MES-PrEP”)

We will build upon existing infrastructure to adapt the 2 components of the intervention (MES-PrEP and MTM) based on responses to interviews. We will use interviews in Phase 1 to adapt the 2-session intervention to promote PrEP. The adaptation process of the Motivational Enhancement System for Adherence will involve two steps: (1) initial programming of sessions of PrEP uptake and adherence intervention in collaboration with the YAB and (2) evaluation of cultural acceptability and intervention content via the YAB and beta-testing. The YMSM will complete 2 MES-PrEP sessions over 1 month.

##### Developing and Programming MTM

We will use automated, bidirectional (“two-way”) MTM via LINE as a tool to support PrEP initiation and adherence [39]. MTM will be developed using information from interviews in Phase 1. Motivational statements promoting PrEP use will be generated from each theme and grouped into one of the 2 MTM libraries—PrEP initiation or PrEP adherence library. Participants will respond to MTM using multiple-choice or open-ended responses, alternated to avoid fatigue. MTM will be sent daily for 1 month, followed by weekly for an additional 5 months (months 2-6) at a time chosen by the participant to promote autonomy.

##### Beta-Testing of MES-PrEP and MTM

The MES-PrEP system will be beta-tested among 10 HIV-negative Thai YMSM. The youth will complete 2 MES-PrEP sessions over 1 month. Each session will be videotaped. We will also assess the MTM system functionality with the same 10 participants who will receive daily MTM between MES-PrEP sessions, using the 2 message libraries. YMSM on PrEP will receive messages from the PrEP adherence library; PrEP-naïve YMSM will receive messages from the PrEP initiation library. Participants will be asked to respond to the prompt(s). After 24 hours of sending the first text message, the staff will call the participant to check if they have encountered any issues with MTM. A brief face-to-face interview will be conducted to (1) identify and correct any technical or other issues encountered in delivering MTM and MES-PrEP and (2) solicit input on how to refine MTM and MES-PrEP delivery and content.

#### Phase 3: Pilot RCT Trial

**Figure 1:**
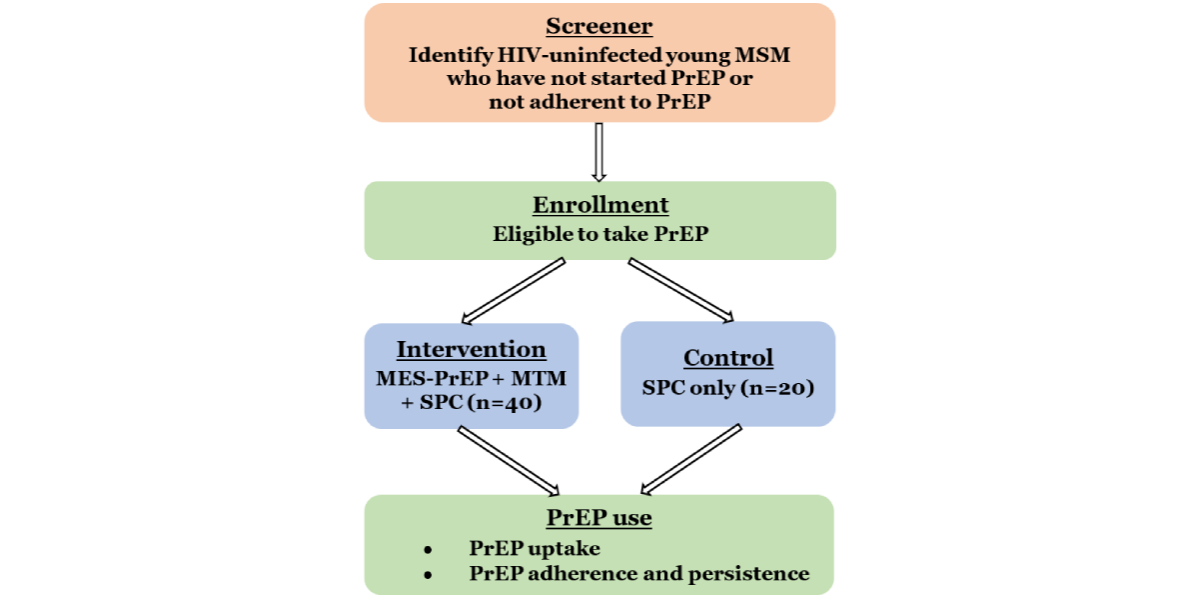
Pilot randomized controlled trial schema. MES-PrEP: Motivational Enhancement System for Pre-Exposure Prophylaxis; MSM: men who have sex with men; MTM: motivational text messaging; PrEP: pre-exposure prophylaxis; SPC: standard PrEP counseling.

We will launch a pilot RCT (registered with ClinicalTrials.gov registry: NCT05243030) to evaluate the feasibility, acceptability, and preliminary efficacy of MES-PrEP and MTM to increase PrEP uptake and adherence among Thai YMSM. The design is shown in Figure 1. We will enroll 120 YMSM (60 YMSM on PrEP and 60 PrEP-naïve YMSM) from the 2 HIV clinics. The 60 PrEP-naïve YMSM are those who are at high risk and eligible for PrEP but have not decided to start PrEP. We anticipate enrollment of 20 participants per month (for 6 months). Participants will be randomized to receive MES-PrEP and MTM (intervention) versus standard PrEP counseling (control) in a 2:1 ratio, stratified by current use of PrEP (PrEP naive vs PrEP users). The 2:1 allocation was chosen to maximize the collection of feasibility and acceptability data among intervention participants. Separate randomization for YMSM who are currently on PrEP and YMSM who are not starting PrEP will be computer-generated using Qualtrics. The primary outcome will be acceptability, feasibility, as well as PrEP uptake and adherence assessed through Computer-Assisted Self-Interview and Dried Blood Spot testing.

### Study Sample

YMSM will be eligible based on inclusion criteria consistent with the Centers for Disease Control and Prevention and Thailand National HIV/AIDS Treatment and Prevention Guidelines [40,41], as follows: (1) age between 16 and 25 years; (2) men who report sex with men in the past 12 months; (3) laboratory-confirmed HIV-negative status; (4) self-reported evidence of being at risk for HIV acquisition, including one of the following in the past 6 months: having sex with an HIV-positive partner, having anal sex without using a condom, being diagnosed with a sexually transmitted infection, or having any illicit drug use (eg, amphetamine-type stimulants); (5) able to understand, read, and speak Thai; and (6) either have not started PrEP (group 1: PrEP naive) or currently on PrEP but not adherent to PrEP (taking ≤3 pills/week) in the past month (group 2: PrEP users). Exclusion criteria are as follows: (1) in a mutually monogamous relationship of >6 months with a partner who recently tested negative for HIV, (2) a serious cognitive or psychiatric problem that would compromise the ability to provide informed consent, and (3) currently enrolled in another HIV intervention study.

### Sample Size of Trial

We use PrEP adherence as a calculating variable, as it is a primary outcome. Using the G*power 3.2 program (2-tailed *t* test: “difference between 2 independent means” procedure), a set effect size of 0.80 (large), α=.05, power=0.80, and an allocation ratio of 2:1, the calculated sample size is 58, with 39 participants in the intervention and 19 in the control group. To detect a medium effect size in PrEP adherence (set effect size 0.50), a sample of 144 is required (96 participants in the intervention and 48 in the control group). Our proposed pilot RCT is not an efficacy study, and thus, it is not able to achieve full statistical power (to detect a small-medium effect size).

### Recruitment and Enrollment Procedures

Young male clients presenting at the 2 study clinics will be prescreened by the clinic service staff based on the study’s eligibility criteria. Potential participants will be referred to trained research staff for more information and the informed consent process. After providing verbal informed consent, the research staff will determine if the youth meets the inclusion criteria. Those who have an HIV-negative status and meet the other inclusion criteria will be invited to join the study. Clinic staff will facilitate the PrEP evaluation, including anti-HIV testing. Those found to have a confirmed HIV-positive status will undergo an evaluation for antiretroviral therapy by clinic staff (standard of care will be provided for HIV-infected youth). The screening visit, enrollment, and baseline assessment should take place on the same day, or failing that, within 1 week. After signed consent is obtained, participants will be asked to provide a phone number or email address and contact information for a family member or friend who can be called in case the participant cannot be reached by phone, email, or text.

### Ethics Approval

This protocol was reviewed and approved by the institutional review boards at the University of Massachusetts Chan Medical School in the United States (H00023523) and Chulalongkorn University in Thailand (Med Chula IRB 919/2564).

## Results

Recruitment for this study began in January 2022 for phase 1. Qualitative interviews were completed with 30 YMSM and 5 clinical providers in May 2022. Qualitative data analysis has been completed, and the results were used to inform intervention adaptation and refinement. In phase 2, our team developed 2 motivational text message libraries, which contain nearly 100 motivational text messages; these messages were reviewed by the YAB and the investigator team for improvement and refinement. Our team also developed a 2-session intervention (MES-PrEP) to promote PrEP uptake and adherence using the Computerized Intervention Authorizing Software (CIAS 3.0; Steven Ondersma). Individual YAB members completed one-on-one beta-testing of the intervention components and provided additional feedback to guide refinement. Phase 3, the pilot feasibility and acceptability trial, began in July 2023. The findings will be disseminated to stakeholders and communities of interest using peer-reviewed journals, academic conferences, and other communication channels.

## Discussion

### Expected Outcomes

We anticipate developing 2 integrated mobile interventions to improve PrEP uptake and adherence among YMSM in Thailand. YMSM are among the most at-risk groups for HIV transmission, yet PrEP use has been low, and adherence is often inadequate. Combining MES-PrEP and MTM may help increase the uptake of and sustained adherence to PrEP among HIV-negative Thai YMSM. We will evaluate the feasibility, acceptability, and preliminary efficacy of an mHealth intervention combining MES-PrEP and MTM. Based on our theoretical models of behavior change, we anticipate that the mHealth intervention will be found to be effective, but this is yet to be determined. Our study addresses major challenges related to PrEP use. If successful, this study will be among the first to identify an mHealth intervention for YMSM in Thailand toward improving PrEP use. Our research is integrated within Thailand's national HIV prevention effort. The commitment and ongoing involvement of the Institute of HIV Research and Innovation at a national level will allow this research to serve as a global model for HIV prevention among at-risk groups, especially for low- and middle-income countries.

### Planned Next Steps

The project will be conducted to develop an integrated technology-based intervention for HIV-negative Thai YMSM and prepare for a future large-scale RCT. For a fully powered project, we would propose a multisite study to test the efficacy of MES-PrEP and MTM. We will adopt a type-2 effectiveness-implementation design with dual testing of the intervention’s effectiveness in achieving the desired outcomes across the PrEP cascade. Additionally, we will gather information on the implementation of technology-based interventions, including cost analysis to assess the relative cost of implementing the intervention.

### Conclusions

This study addresses a critical problem (high HIV incidence and low PrEP use) among an at-risk population (YMSM) in Thailand. We are developing 2 potentially synergistic technology-based, theory-driven interventions aimed at maximizing PrEP uptake and sustained PrEP adherence. Even though Thailand was involved in the world’s first PrEP clinical trial (iPrEP) and several PrEP demonstration projects, PrEP uptake and adherence remain low in Thailand. Our study addresses major challenges related to PrEP use. It is responsive to the US Preventive Services Task Force’s recommendations that call for research on methods to increase PrEP uptake and adherence [30]. If successful, this study will pave the way for the successful scale-up of PrEP implementation among MSM in Thailand.

## Appendix

Multimedia Appendix 1 Peer-review reports from the HIV/AIDS Intra- and Inter-personal Determinants and Behavioral Interventions Study Section (HIBI) - Risk, Prevention and Health Behavior Integrated Review Group - Center for Scientific Review (National Institutes of Health, USA).

## Abbreviations

IMB: Information-Motivation-Behavioral Skills Model
MES-PrEP: Motivational Enhancement System for Pre-Exposure Prophylaxis
mHealth: mobile health
MI: motivational interviewing
MSM: men who have sex with men
MTM: motivational text messaging
PrEP: pre-exposure prophylaxis
RCT: randomized controlled trial
RSAT: Rainbow Sky Association of Thailand
YAB: youth advisory board
YMSM: young men who have sex with men

## Data Availability

The data generated during this study are available from the corresponding author upon reasonable request.
