# Supplementary material for: Adapting Effective mHealth Interventions to Improve Uptake and Adherence to HIV Pre-Exposure Prophylaxis Among Thai Young Men Who Have Sex With Men: Protocol for a Randomized Controlled Trial
Source: JMIR Res Protoc. 2023 Sep 4;12:e46435. doi: 10.2196/46435 (PMC10507518; doi:10.2196/46435)
Supplement: Multimedia Appendix 1 [file resprot_v12i1e46435_app1.pdf]

SUMMARY STATEMENT

PROGRAM CONTACT:
Susannah Allison
240-627-3861
allisonsu@mail.nih.gov

(Privileged Communication)

Release Date: 12/05/2020
Revised Date:

Application Number: 1 R34 MH124081-01A1
Formerly: 1R34MH124081-01

Principal Investigators (Listed Alphabetically):

PHANUPHAK, NITTAYA
WANG, BO (Contact)

Applicant Organization: UNIV OF MASSACHUSETTS MED SCH WORCESTER

Review Group: HIBI
HIV/AIDS Intra- and Inter-personal Determinants and Behavioral Interventions Study Section
AIDS - EXP. REV.

Meeting Date: 11/12/2020
Council: JAN 2021
Requested Start: 04/01/2021
RFA/PA: PA20-141
PCC: 9A-ASPA

Project Title: Adapting effective mhealth interventions to improve uptake and adherence of the HIV pre- exposure prophylaxis (PrEP) in Thai young MSM
SRG Action: Impact Score:33 Percentile:12 +
Next Steps: Visit https://grants.nih.gov/grants/next_steps.htm
Human Subjects: 30-Human subjects involved - Certified, no SRG concerns
Animal Subjects: 10-No live vertebrate animals involved for competing appl.
Gender: 3A-Only men, scientifically acceptable
Minority: 5A-Only foreign subjects, scientifically acceptable
Age: 6A-Children and Adults, scientifically acceptable

Project Year	Direct Costs Requested	Estimated Total Cost
1	150,000	223,820
2	150,000	223,820
3	150,000	223,820
TOTAL	450,000	671,459

ADMINISTRATIVE BUDGET NOTE: The budget shown is the requested budget and has not been adjusted to reflect any recommendations made by reviewers. If an award is planned, the costs will be calculated by Institute grants management staff based on the recommendations outlined below in the COMMITTEE BUDGET RECOMMENDATIONS section.

1R34MH124081-01A1 Wang, Bo

RESUME AND SUMMARY OF DISCUSSION: This application proposes to develop and pilot test a computer-based motivational interview intervention for HIV+ youth combined with a Motivational Interactive Text Messaging that focuses on PrEP uptake and adherence that will be adapted for use with Thai HIV-negative MSM youth. The new intervention, Motivational Enhancement System for PrEP Uptake and Adherence (MES-PrEP), will be pilot tested with 120 HIV-negative young MSM (60 PrEP naïve and 60 PrEP users) that are randomly assigned 2:1 to the intervention or to standard PrEP counseling. PrEP uptake and adherence to PrEP will be the primary outcomes. Given the high rates of HIV among Thai MSM and very low rates of PrEP uptake by these men, the proposed intervention has the potential to have a significant public health impact. The study team is strong and has the expertise to carry out the proposed study. The team has been highly responsive to concerns raised in the prior review of this application. The MPI plan was revised and a team member with overlapping expertise was removed. The team included the Socioecological Model that addresses social and structural barriers and clarified a number of issues including the description of the text messaging component. Finally, the number of men participating in the in-depth interviews in phase one was nearly doubled. The committee discussed the potential impact of long-acting injectable formulations on the proposed intervention and there was also some concern that preliminary data presented in the application was not yet published. The application of the ADAPT-ITT was also questioned. The majority of the committee felt that these remaining concerns were acceptable for a R34 application and, therefore, the committee's overall enthusiasm for the resubmission remained high.

DESCRIPTION (provided by applicant): Young men having sex with men (YMSM) are the fastest growing HIV+ population worldwide. Thailand has the highest adult HIV seroprevalence in Asia; 28.6% of men having sex with men in Bangkok are HIV+. The HIV incidence rate among YMSM in Bangkok is 12.2 per 100 person-years. Effective prevention strategies are urgently needed to prevent HIV transmission in this high-risk population. Pre-exposure prophylaxis (PrEP) is an efficacious HIV prevention strategy recommended by the CDC and WHO for all at-risk individuals, including HIV-negative MSM. PrEP is highly effective when taken as prescribed, but PrEP utilization has been low and adherence is often inadequate. To address these challenges among Thai YMSM, we propose to develop a technology facilitated intervention combining Motivational Enhancement System for PrEP Uptake and Adherence (MES-PrEP) and Motivational Interactive Text Messaging (MTM) to increase uptake of and sustained adherence to PrEP among HIV-negative, Thai YMSM. MTM will be programmed and tested to send two-way motivational messages to promote PrEP uptake and adherence. Our primary aim is to develop and pilot test MES-PrEP and MTM and associated protocols and to assess preliminary evidence of the effect of the technology facilitated intervention. The proposed study includes 3 phases. Phase I includes in-depth interviews with 30 HIV-negative Thai YMSM to explore barriers and facilitators of PrEP initiation and adherence to inform intervention content. Phase II consists of adapting and beta testing MES-PrEP and MTM for functionality and feasibility using 10 HIV-negative Thai YMSM. In Phase III, we will conduct a pilot randomized controlled trial to evaluate the feasibility, acceptability, and preliminary efficacy of MAS-PrEP and MTM to increase PrEP uptake and adherence among Thai YMSM. We will enroll 60 HIV-negative Thai YMSM who have not started PrEP and 60 YMSM who are on PrEP but not adherent to it. Participants will be randomized 2:1 to received MES-PrEP and MTM (n=40) or Standard PrEP Counseling (n=20). Feasibility and acceptability of the intervention will be assessed through paradata of usage patterns and the System Usability Scale. Preliminary impact will be assessed by evaluating the proportion of PrEP initiation and level of adherence to PrEP. Participants will complete the assessments at baseline and 1, 3, and 6 months post- intervention. Biomarkers of adherence to PrEP and HIV/STI will be collected. Upon project completion, we shall have developed a highly innovative mHealth intervention to support MSM using PrEP which will be ready for testing in a larger efficacy trial.

PUBLIC HEALTH RELEVANCE: Pre-exposure prophylaxis (PrEP) is highly effective in preventing HIV infection, yet rates of initiation and adherence are low and discontinuation rates are high in Thai YMSM, a high-risk population with HIV prevalence rate as high as 28.6%. This project will develop a technology facilitated intervention combining Motivational Enhancement System for PrEP Uptake and Adherence (MES-PrEP) and Motivational Interactive Text Messaging (MTM) to increase uptake of and sustained adherence to PrEP among HIV-negative, Thai YMSM. This study has potential to pave the way to the successful scale-up of PrEP implementation in Thailand.

CRITIQUE 1

Significance: 3
Investigator(s): 1
Innovation: 1
Approach: 3
Environment: 1

Overall Impact*:* This is a resubmission of an R34. MSM are 20 times as likely to be living with HIV than the general population. HIV prevalence among MSM in Thailand is nearly 30% with a particularly rapid rate of increase among youth 15-21 years. PrEP uptake and adherence is low among MSM. Barriers to initiating or staying on PrEP include low self-perceived risk, concerns of side-effects, drug use and HIV stigma. The goal of the application is to develop and pilot an intervention comprising a two-session computer-based motivational interview and motivational interactive text messaging to increase PrEP uptake and adherence among Thai HIV-negative MSM youth. Aim 1 will comprise 30 in-depth interviews with young MSM on PrEP and PrEP-naive to understand barriers and facilitators of PrEP. Aim 2 is to pilot test the adapted MES-PrEP among 120 young MSM (1/2 PrEP naïve and ½ on PrEP) with 1-month follow-up. Primary outcomes are PrEP uptake and adherence. After the first computer-based MI session, participants will receive daily text messages. After the second session, one month after the first, participants will receive weekly text messages until the last follow-up at month 6. Assessments will be at baseline, 1, 3 and 6-month interviews and comprise a survey as well as biomarkers that include DBS for PrEP adherence, STI and drug testing. The resubmission is highly responsive to the reviewers' comments that include increasing the sample size from 16 to 30 in-depth interviews in Aim 1, adding the socioecological model to incorporate structural and social-level factors, adding detail to the adaptation of the MESA platform, clarification of the size of the effect of MESA on ART adherence, and changing the exit interview from 6 to 1 month. Overall this is a very strong application led by an excellent investigative team. A moderate concern centers around the reality of addressing structural and social interventions through this intervention despite the addition of the SEM in the conceptual framework. As such, the inclusion of the SEM model seems more of a checking of a box rather than a commitment to addressing social and structural barriers. A few additional minor concerns are detailed below.

1. Significance:

Strengths

- The rapidly expanding HIV epidemic among YMSM globally and in Thailand in particular (with an incidence of 12/100 PY in Bangkok) and the low rates of PrEP uptake and adherence underscore the need for interventions to increase PrEP use among HIV-negative young MSM.
- Truvada is available but underutilized in Thailand. An estimated 40% are currently on PrEP, and only 9.3% of Thai MSM who were offered PrEP started it.

- Development of a technology-based intervention offers advantages in reach, cost, anonymity and therefore scalability.
- 90% of YMSM in Thailand had a cell phone in 2014, and that number has likely increased.

Weaknesses

- Although the application now incorporates SEM into the conceptual framework, it's not clear how social and structural elements will actually be addressed in this intervention. The application mentions depression and stigma (both experienced at the individual level) as outcomes without explaining how these will be addressed from a societal or structural perspective.
- Although the application states that it is unlikely that LAI will be approved in Thailand, it's important to note the long-term vision for the intervention: how would the relevance of this intervention be affected by the presence of long-acting injectables in the Thai PrEP landscape. Could the intervention be adapted to LAI? Or would it be irrelevant for those on LAI?

2. Investigator(s):

Strengths

- Dr. Wang (PI) has a strong track record of HIV research in Thailand, including the adaptation of the MI-based intervention for Thai youth living with HIV. Dr. Phanuphak (MPI) is Chief of Prevention has extensive experience with clinical trials (including HPTN) and has 3 ongoing PrEP trials.
- The team is well rounded with Dr. Boudreaux providing expertise in HIV prevention interventions; Dr. MacDonnell provides expertise in development and pediatric psychology, Dr. Naar has expertise in MI and Dr. Stanton on intervention research. Dr. Sadasivam is a computer engineer with extensive expertise in developing technology-facilitated behavioral health interventions.
- Dr. Naar has used CIAS to develop an interactive MI session for sexual risk reduction for youth living with HIV.
- The MPI plan includes a reasonable resolution to potential conflicts (Dr. Stanton will resolve if a solution cannot be found). The MPIs have a history of collaboration as reflected on their publications.

Weaknesses

- None noted by reviewer

3. Innovation:

Strengths

- Combining a computer-based intervention and text messaging is innovative and particularly relevant to YMSM who are hard to reach but technologically savvy and receptive to this type of format.
- The ability to tailor both technology-based components of the intervention is novel. Both are interactive in the sense that content of the sessions and messages are dependent on prior responses from the participant.
- The addition of biomarkers is not new, but supports the validity of outcome measures.

Weaknesses

- None noted by reviewer

4. Approach:

Strengths

- The ubiquitous nature of cell phones in Thailand make this a feasible intervention for YMSM.
- The steps to adapt MESA and develop MES-PrEP and MTM (following ADAPT-ITT) are reasonable, thoughtful and thorough (Aim 1);
- Outcome measure of feasibility and acceptability are appropriate along with preliminary efficacy outcomes: PrEP uptake and adherence.
- The addition of SEM expands the scope beyond the individual and incorporates social and structural context into the study.
- DBS for PrEP adherence, STI testing and drug testing are biomarkers that strengthen the validity of self-reported outcomes
- Use of CIAS technology that is flexible and easily programmed by clinicians for MI sessions. The latter feature makes it scalable and sustainable over time.
- Both computer-based components are tailored to the individual participants: MI is interactive—each session is unique based on participant's responses; text messages are based on content from individual MES-PrEP including readiness to take PrEP, and PrEP adherence.
- The Youth Advisory groups and clinical providers will both be involved in developing content of the MI sessions and text messages ensuring content that is both population and clinically appropriate.
- Preliminary studies support this study: with MESA reducing viral load among youth living with HIV on ART in a large RCT and high levels of acceptability among participants.

Weaknesses

- While SEM is added to the framework, it's unclear how the structural concepts themselves will be operationalized.
- Drug use is mentioned as a barrier to PrEP uptake and adherence but not discussed in the application (type of drugs, prevalence, how the intervention will address it).
- Acceptability and feasibility are measured from the perspective of participants; however it would be helpful for future studies and scale-up to understand acceptability and feasibility from the implementers perspectives including providers and clinicians who have may have to re-program the platforms based on evolving information.
- STIs are secondary outcome measures but the rationale for including them are never addressed.
- It is unclear why TGW or TGM youth are not included in this study.

5. Environment:

Strengths

- University of Massachusetts Medical School and the Thai Red Cross AIDS Research Center (the Men's Health Clinic and Tangerine Community Health Center) are outstanding research

environments to oversee the administrative and operational aspects of this study respectively. The Thai Red Cross AIDS Research Center is an HPTN site with extensive experience with complicated and rigorous clinical trials that are used for FDA filings.

Weaknesses

- None noted by reviewer

Study Timeline:

Strengths

- The timeline is reasonable.

Weaknesses

- None noted by reviewer

Protections for Human Subjects:

Acceptable Risks and/or Adequate Protections

Data and Safety Monitoring Plan (Applicable for Clinical Trials Only):

Acceptable

Inclusion Plans:

- Sex/Gender: Distribution not justified scientifically
- Race/Ethnicity: Distribution justified scientifically
- For NIH-Defined Phase III trials, Plans for valid design and analysis: Not applicable
- Inclusion/Exclusion Based on Age: Distribution justified scientifically
- unclear why trans/nonbinary persons are not included

Vertebrate Animals:

Not Applicable (No Vertebrate Animals)

Biohazards:

Not Applicable (No Biohazards)

Resubmission:

- Highly responsive to prior review

Resource Sharing Plans:

Acceptable

Authentication of Key Biological and/or Chemical Resources:

Not Applicable (No Relevant Resources)

Budget and Period of Support:

Recommend as Requested

CRITIQUE 2

Significance: 2
Investigator(s): 1
Innovation: 1
Approach: 3
Environment: 2

Overall Impact: The purpose of this revised R34 is to adapt an MI-based electronic agent intervention to deliver a 2-session PrEP uptake and adherence intervention for young (16-25) Thai men who have sex with men in Bangkok. This is a well-written and highly responsive revised application. The study team is highly experienced and the environment is strong. The scientific premise of the proposed intervention is strong and the combination of the electronic MI-based agent and tailored text messages is innovative. There were some minor weaknesses in the approach, but overall the description of the phases of the study (including the interviews in Phase 1, adaptation in Phase 2, and the pilot RCT in Phase 3) is very thorough. Overall, this is a strong application with several addressable minor weaknesses.

1. Significance:

Strengths

- Rates of HIV infection among young men who have sex with men (YMSM) are disproportionately high and rates of HIV among Thai MSM are estimated to be 20-30%. There is a clear need for innovative interventions addressing HIV in Thai MSM.
- PrEP is an effective prevention tool, but willingness to take PrEP among Thai MSM is low (~40%) and only 9.3% of Thai MSM who were offered PrEP started it. There is clearly a need for more robust PrEP interventions that target uptake and maintenance on PrEP in Thailand.
- Smartphone use in Thailand is very high and therefore the SMS component of the proposed intervention may be scalable if shown to be effective.
- The ability for the computer software that runs the agent to be tailored easily to different contexts may make it highly scalable.
- The scientific premise of the study is strong. The investigators directly addressed concerns about data speed, the potential impacts of approval of long-acting injectables, and how they would better understand mechanisms preventing uptake and adherence.

Weaknesses

- A relatively minor concern is that the MI agent can only be viewed in the clinic and therefore this may limit its potential scalability.

2. Investigator(s):

Strengths

- Dr. Wang (PI/PD) is an epidemiologist and has been involved for a decade in HIV prevention and treatment research in Thailand. He was involved with adapting MI-based intervention for HIV-positive Thai youth, which is the basis of the proposed study. He is well qualified to serve as an MPI for the proposed work.
- Dr. Phanuphak (MPI) is the Chief of Prevention and the Chief of Search at the TRCARC) and has extensive experience in conducting clinical research, including overseeing 3 PrEP studies in Thailand currently.
- Dr. MacDonell (co-I) is a developmental and pediatric psychologist and has expertise in HIV behavioral intervention and the use of technologies for MI-based intervention.
- Dr. Sadasivam (co-I) is a computer engineer with expertise in technology-facilitated behavioral intervention.
- Dr. Rongkavilit (co-I) is a physician and clinical researcher with expertise in HIV medicine and behavioral research in Thailand.
- The team is supported by expert consultants in MI (Dr. Naar) and international HIV intervention research (Dr. Stanton).
- Some of the team members have a history of collaboration.

Weaknesses

- None noted by reviewer

3. Innovation:

Strengths

- The use of an electronic agent to deliver MI is innovative in the context of PrEP uptake and adherence among Thai MSM
- The pairing of the MI agent with regular 2-way text messaging is innovative.

Weaknesses

- None noted by the reviewer.

4. Approach:

Strengths

- Well articulated study aims.
- The theoretical grounding of the intervention in IMB and MI is well described and is consistently applied throughout the intervention and assessment.
- The use of the ADAPT-ITT model for adaptation is a strength.
- The study builds off of prior work by this study team demonstrating their ability to successfully carry out similar projects.
- Providing mobile devices to participants who may not have one is a strength.
- Clear description for how interviews in Phase I will inform intervention adaptation.
- The adaptation process is thoroughly described.
- The control condition is appropriate and the 2:1 randomization was justified.

- Clear plan for linking participants who test positive for HIV or syphilis to care.
- The scientific rigor of the proposed study is high.

Weaknesses

- The section on the PrEP cascade that seemed to imply that the stages of change model was also driving intervention development.
- It was unclear whether participants had to opt into receiving text messages after the first month.
- Only interviewing 20 participants for the exit interviews is a missed opportunity to get more feedback on the intervention from more participants.
- The quantitative acceptability measures could be more robust, as the SUS is fairly narrow in what it assesses.
- It wasn't clear where some of the measures (e.g., Decisional Balance for PrEP Use) were sourced.

5. Environment:

Strengths

- The University of Massachusetts Medical School – which houses the Population and Quantitative Health Sciences Department – is a strong research environment to oversee the operational and financial aspects of the proposed study.
- The Institute of HIV Research and Innovation has substantial resources to oversee the day-to-day operations of the proposed study in Thailand.
- UCSF and Wayne state are appropriate environments to oversee the work being done by the co-Is at those institutions.

Weaknesses

- The recruitment clinics seem like they may be excellent sources for participants, however the recruitment and retention plan is not particularly well articulated.

Study Timeline:

Strengths

- The timeline is tight, but the investigators are highly experienced.
- Study team organization is clear.

Weaknesses

- The recruitment and retention plan are not very specific about procedures that will be used and the success of those procedures in prior studies.

Protections for Human Subjects:

Acceptable Risks and/or Adequate Protections

- Risk adequately described and ways to mitigate those risks considered.

Data and Safety Monitoring Plan (Applicable for Clinical Trials Only):

Acceptable

- Identification and addressing AEs and SAEs described.

Inclusion Plans:

- Sex/Gender: Distribution justified scientifically
- Race/Ethnicity: Distribution justified scientifically
- For NIH-Defined Phase III trials, Plans for valid design and analysis: Not applicable
- Inclusion/Exclusion Based on Age: Distribution justified scientifically
- Inclusion of 16-17 year old children. Enrollment limited to men, with no discussion of whether transgender men will be included. Could be strengthened by rationale for excluding transwomen. Race/ethnicity will reflect local population.

Vertebrate Animals:

Not Applicable (No Vertebrate Animals)

Biohazards:

Not Applicable (No Biohazards)

Resubmission:

- Highly responsive to prior critiques.

Applications from Foreign Organizations:

- Notes how this study, if successful, can serve as a global model for HIV prevention of at-risk groups in LMICs.

Resource Sharing Plans:

Acceptable

Authentication of Key Biological and/or Chemical Resources:

Not Applicable (No Relevant Resources)

Budget and Period of Support:

Recommend as Requested

CRITIQUE 3

Significance: 2
Investigator(s): 2
Innovation: 4

Approach: 5
Environment: 2

Overall Impact: Applicants propose to adapt existing interventions to support PrEP uptake and adherence in young Thai MSM. This is a high-risk population, and there is a need for scalable interventions to support improved uptake. The proposal has several strengths, including a strong clinical setting, a strong research team, and a well-defined set of outcomes from the pilot RCT. Enthusiasm is somewhat limited by a suggestion that clinicians could modify the intervention platform, which would decrease rigor; by preliminary data on MESA that were presented a year and a half ago but never published; and by a suggestion that some steps of the ADAPT-ITT approach will be skipped through a preemptive choice of the intervention to modify. The proposal to use a well-understood framework to guide the selection and adaptation of the intervention but preempt that framework to choose the investigators' own intervention without a systematic process compromises the value of using the framework.

1. Significance:

Strengths

- Thai MSM are at high risk for HIV infection
- Scalable tools to increase PrEP uptake and adherence are a critical need
- Adapting existing approaches is a time-saving approach to develop culturally appropriate intervention approaches

Weaknesses

- Data on low uptake and persistence presented are mostly US data; unclear if these are the same critical issues in Thai MSM

2. Investigator(s):

Strengths

- Dr. Wang is well qualified to lead the proposed work
- Dr. Phanuphak is well qualified to serve as a dual PI and oversee research activities
- Consultants are well positioned to support the study team.

Weaknesses

- None noted by reviewer

3. Innovation:

Strengths

- The intervention with Thai YMSM is innovative

Weaknesses

- Use of DBS to measure PrEP adherence is not innovative

4. Approach:

Strengths

- Preliminary data on MI are compelling
- Appropriately phased plan for development of content, production of intervention components and pilot RCT
- YAB will provide critical input into the development of the intervention
- Use of ADAPT-ITT model is appropriate and provides a good framework for the adaptation
- The development of SMS content from the interview context is a strong approach
- Mediation analyses using SEM are important supplemental analyses

Weaknesses

- The description of the MI platform mentions that the software might be reprogrammed by researchers, clinical staff or clinicians to address different behaviors or contexts. But it is not clear how decisions about changes to the software would be made or documented, and how the impact of the intervention would be assessed if the intervention is changed (or changeable) by clinicians during the study. If it is the intention that the intervention could be expanded when new therapies are approved (e.g., long acting injectable PrEP), this could be a plus, but as described, having clinicians or clinical staff change the app to target new health behaviors is problematic.
- The preliminary data presented on MESA are from a small number of participants, and data on efficacy presented are from an abstract presented a year and half ago and not subsequently published (based on a search conducted for recent publication at the time of review). It is speculative to rely on data from a conference presentation that has not been peer reviewed and is not available for review to endorse a major component of the intervention approach. Although preliminary data are not required for this mechanism, MESA is described as a successful intervention, and having stronger data to support this assertion would make the proposal stronger.
- ADAPT-ITT suggests relying on data from phase 1 to choose which intervention to adapt, but the proposal states that MESA has already been selected based on prior work with MSM. If the framework is to be used meaningfully, it seems odd to go in with a decision made about what the best intervention to adapt is, without following the earlier steps or considering other interventions for adaptation. If the applicants want to use a subset of the ADAPT-ITT process by skipping steps 1-2, this should be stated.
- The stages of change model seems to be an awkward addition conceptually to the PrEP cascade discussion, and really isn’t integrated into the rest of the proposal.
- The SUS is a very limited assessment of usability in terms of identifying actionable revisions; qualitative data would strengthen the actionable assessment usability and how to improve it.

5. Environment:

Strengths

- The TRC and RSAT clinics are very strong settings for the proposed research
- UMass, Wayne State and UCSF are strong institutional settings

Weaknesses

- None noted by reviewer

Study Timeline:

Strengths

- Clear measures for acceptability and feasibility are proposed
- Biological measure for PrEP adherence is a strength, and detailed information about the proper storage and handling of the specimens to assure accuracy of testing

Weaknesses

- None noted by reviewer

Protections for Human Subjects:
Acceptable Risks and/or Adequate Protections

Data and Safety Monitoring Plan (Applicable for Clinical Trials Only):
Acceptable

Inclusion Plans:

- Sex/Gender: Distribution justified scientifically
- Race/Ethnicity: Distribution justified scientifically
- For NIH-Defined Phase III trials, Plans for valid design and analysis: Not applicable
- Inclusion/Exclusion Based on Age: Distribution justified scientifically

Vertebrate Animals:
Not Applicable (No Vertebrate Animals)

Biohazards:
Not Applicable (No Biohazards)

Resource Sharing Plans:
Acceptable

Authentication of Key Biological and/or Chemical Resources:
Not Applicable (No Relevant Resources)

Budget and Period of Support:
Recommend as Requested

THE FOLLOWING SECTIONS WERE PREPARED BY THE SCIENTIFIC REVIEW OFFICER TO SUMMARIZE THE OUTCOME OF DISCUSSIONS OF THE REVIEW COMMITTEE, OR REVIEWERS' WRITTEN CRITIQUES, ON THE FOLLOWING ISSUES:

PROTECTION OF HUMAN SUBJECTS: ACCEPTABLE

INCLUSION OF WOMEN PLAN: ACCEPTABLE

INCLUSION OF MINORITIES PLAN: ACCEPTABLE

INCLUSION ACROSS THE LIFESPAN: ACCEPTABLE

COMMITTEE BUDGET RECOMMENDATIONS: The budget was recommended as requested.

Footnotes for 1 R34 MH124081-01A1; PI Name: Wang, Bo

+ Derived from the range of percentile values calculated for the study section that reviewed this application.

NIH has modified its policy regarding the receipt of resubmissions (amended applications).See Guide Notice NOT-OD-18-197 at https://grants.nih.gov/grants/guide/notice-files/NOT-OD-18-197.html. The impact/priority score is calculated after discussion of an application by averaging the overall scores (1-9) given by all voting reviewers on the committee and multiplying by 10. The criterion scores are submitted prior to the meeting by the individual reviewers assigned to an application, and are not discussed specifically at the review meeting or calculated into the overall impact score. Some applications also receive a percentile ranking. For details on the review process, see http://grants.nih.gov/grants/peer_review_process.htm#scoring.

MEETING ROSTER

HIV/AIDS Intra- and Inter-personal Determinants and Behavioral Interventions Study Section
Risk, Prevention and Health Behavior Integrated Review Group
CENTER FOR SCIENTIFIC REVIEW
HIBI
11/12/2020 - 11/13/2020

Notice of NIH Policy to All Applicants: Meeting rosters are provided for information purposes only. Applicant investigators and institutional officials must not communicate directly with study section members about an application before or after the review. Failure to observe this policy will create a serious breach of integrity in the peer review process, and may lead to actions outlined in NOT-OD-14-073 at https://grants.nih.gov/grants/guide/notice-files/NOT-OD-14-073.html and NOT-OD-15-106 at https://grants.nih.gov/grants/guide/notice-files/NOT-OD-15-106.html, including removal of the application from immediate review.

CHAIRPERSON(S)

VREEMAN, RACHEL CHRISTINE, MD
PROFESSOR
DEPARTMENT OF HEALTH SYSTEM DESIGN
AND GLOBAL HEALTH
ICAHN SCHOOL OF MEDICINE AT MOUNT SINAI
NEW YORK CITY, NY 10029

MEMBERS

BOGART, LAURA M, PHD *
SENIOR BEHAVIORAL SCIENTIST
RAND CORPORATION
SANTA MONICA, CA 90407-2138

BUTLER, LISA MICHELLE, PHD
ASSOCIATE RESEARCH PROFESSOR
INSTITUTE FOR COLLABORATION ON HEALTH,
INTERVENTION, AND POLICY
UNIVERSITY OF CONNECTICUT
STORRS, CT 06269

COMULADA, WARREN SCOTT, DRPH
ASSOCIATE PROFESSOR
DEPARTMENT OF PSYCHIATRY
AND BIOBEHAVIORAL SCIENCES
SCHOOL OF PUBLIC HEALTH
UNIVERSITY OF CALIFORNIA, LOS ANGELES
LOS ANGELES, CA 90024

FLEMING, PAUL JOSEPH, PHD *
ASSISTANT PROFESSOR
DEPARTMENT OF HEALTH BEHAVIOR
AND HEALTH EDUCATION
SCHOOL OF PUBLIC HEALTH
UNIVERSITY OF MICHIGAN
ANN ARBOR, MI 48109-2029

FRYE, VICTORIA, PHD *
MEDICAL PROFESSOR
DEPARTMENT OF COMMUNITY HEALTH
AND SOCIAL MEDICINE
SCHOOL OF MEDICINE
THE CITY UNIVERSITY OF NEW YORK
NEW YORK, NY 10035

GLASMAN, LAURA R, PHD *
ASSISTANT PROFESSOR
DEPARTMENT OF PSYCHIATRY AND BEHAVIORAL MEDICINE
CENTER FOR AIDS INTERVENTION
MEDICAL COLLEGE OF WISCONSIN
MILWAUKEE, WI 53223

GO, VIVIAN F, PHD *
ASSOCIATE PROFESSOR
DEPARTMENT OF HEALTH BEHAVIOR
GILLINGS SCHOOL OF GLOBAL PUBLIC HEALTH
UNIVERSITY OF NORTH CAROLINA
CHAPEL HILL, NC 27599

GRAHAM, SUSAN MARIE, MD, PHD
ASSOCIATE PROFESSOR
DIVISION OF ALLERGY AND INFECTIOUS DISEASES
DEPARTMENTS OF MEDICINE AND GLOBAL HEALTH
SCHOOL OF MEDICINE
UNIVERSITY OF WASHINGTON
SEATTLE, WA 98104

GROV, CHRISTIAN, PHD
PROFESSOR AND CHAIR
DEPARTMENT OF COMMUNITY HEALTH
AND SOCIAL SCIENCES
SCHOOL OF PUBLIC HEALTH AND HEALTH POLICY
CITY UNIVERSITY OF NEW YORK
NEW YORK, NY 10027

HANSEN, NATHAN B, PHD
DEPARTMENT HEAD AND PROFESSOR
DEPARTMENT OF HEALTH PROMOTION AND BEHAVIOR
COLLEGE OF PUBLIC HEALTH
UNIVERSITY OF GEORGIA
ATHENS, GA 30602

HORVATH, KEITH JOSEPH, PHD
ASSOCIATE PROFESSOR
DEPARTMENT OF CLINICAL PSYCHOLOGY
SAN DIEGO STATE UNIVERSITY
SAN DIEGO, CA 92120

IWELUNMOR, JULIET, PHD *
ASSOCIATE PROFESSOR
DEPARTMENT OF BEHAVIORAL SCIENCE
AND HEALTH EDUCATION
COLLEGE FOR PUBLIC HEALTH AND SOCIAL JUSTICE
SAINT LOUIS UNIVERSITY
ST. LOUIS, MO 63104

KIPKE, MICHELE D, PHD
PROFESSOR
DEPARTMENT OF PEDIATRICS AND PREVENTIVE MEDICINE
KECK SCHOOL OF MEDICINE
UNIVERSITY OF SOUTHERN CALIFORNIA
LOS ANGELES, CA 90027

LEHMAN, WAYNE E K, PHD *
SENIOR RESEARCH SCIENTIST
INSTITUTE OF BEHAVIORAL RESEARCH
TEXAS CHRISTIAN UNIVERSITY
FORT WORTH, TX 76129

LEKAS, HELEN-MARIA, PHD *
ASSOCIATE PROFESSOR
DIVISION OF SOCIAL SOLUTIONS AND SERVICES
RESEARCH
NATHAN KLINE INSTITUTE FOR PSYCHIATRIC RESEARCH
LANGONE SCHOOL OF MEDICINE
NEW YORK UNIVERSITY
NEW YORK, NY 10012

LOVEJOY, TRAVIS IAN, PHD *
ASSOCIATE PROFESSOR
DEPARTMENT OF PSYCHIATRY
SCHOOL OF MEDICINE
OREGON HEALTH AND SCIENCE UNIVERSITY
PORTLAND, OR 97239

LUSENO, WINFRED K, PHD *
RESEARCH SCIENTIST
PACIFIC INSTITUTE FOR RESEARCH AND EVALUATION
CHAPEL HILL, NC 27514

MACKESY-AMITI, MARY ELLEN, PHD *
ASSOCIATE PROFESSOR
DIVISION OF EPIDEMIOLOGY AND BIOSTATISTICS
SCHOOL OF PUBLIC HEALTH
UNIVERSITY OF ILLINOIS, CHICAGO
CHICAGO, IL 60453

MCHENRY, MEGAN SONG, MD *
ASSISTANT PROFESSOR
DIVISION OF PEDIATRIC INFECTIOUS DISEASE
AND GLOBAL HEALTH
SCHOOL OF MEDICINE
INDIANA UNIVERSITY
INDIANAPOLIS, IN 46202

OSTERMANN, JAN, PHD *
ASSOCIATE PROFESSOR
DEPARTMENT OF HEALTH SERVICES POLICY
AND MANAGEMENT
ARNOLD SCHOOL OF PUBLIC HEALTH
UNIVERSITY OF SOUTH CAROLINA
COLUMBIA, SC 29208

RAMSEY, SUSAN E, PHD
ASSOCIATE PROFESSOR
DIVISION OF GENERAL INTERNAL MEDICINE
RHODE ISLAND HOSPITAL
BROWN UNIVERSITY
PROVIDENCE, RI 02903

SSEWAMALA, FRED M, PHD
PROFESSOR
INSTITUTE FOR PUBLIC HEALTH
BROWN SCHOOL
WASHINGTON UNIVERSITY
ST. LOUIS, MO 63130

STOCKMAN, JAMILA KINSHASA, PHD
ASSOCIATE PROFESSOR
DIVISION OF GLOBAL PUBLIC HEALTH
DEPARTMENT OF MEDICINE
SCHOOL OF MEDICINE
UNIVERSITY OF CALIFORNIA, SAN DIEGO
LA JOLLA, CA 92093

SULLIVAN, PATRICK SEAN, PHD
PROFESSOR
DEPARTMENT OF EPIDEMIOLOGY
ROLLINS SCHOOL OF PUBLIC HEALTH
EMORY UNIVERSITY
ATLANTA, GA 30322

THAMES, APRIL D, PHD
ASSOCIATE PROFESSOR
DEPARTMENT OF PSYCHOLOGY
UNIVERSITY OF SOUTHERN CALIFORNIA
LOS ANGELES, CA 90089

TOBIN, KARIN E, PHD
ASSOCIATE PROFESSOR
DEPARTMENT OF HEALTH, BEHAVIOR, AND SOCIETY
BLOOMBERG SCHOOL OF PUBLIC HEALTH
JOHNS HOPKINS UNIVERSITY
BALTIMORE, MD 21205

TURAN, JANET M, PHD
PROFESSOR
DEPARTMENT OF HEALTH CARE ORGANIZATION
AND POLICY
SCHOOL OF PUBLIC HEALTH
UNIVERSITY OF ALABAMA AT BIRMINGHAM
BIRMINGHAM, AL 35294

WEBEL, ALLISON R, PHD
ASSOCIATE PROFESSOR
FRANCIS PAYNE BOLTON SCHOOL OF NURSING
CASE WESTERN RESERVE UNIVERSITY
CLEVELAND, OH 44106

WEISER, SHERI DAWN, MD *
ASSOCIATE PROFESSOR
DEPARTMENT OF MEDICINE
SCHOOL OF MEDICINE
UNIVERSITY OF CALIFORNIA SAN FRANCISCO
SAN FRANCISCO, CA 94110

WINDSOR, LILIANE CAMBRAIA, PHD
ASSOCIATE PROFESSOR
SCHOOL OF SOCIAL WORK
THE UNIVERSITY OF ILLINOIS AT URBANA-CHAMPAIGN
URBANA, IL 61801

MAIL REVIEWER(S)

DODGE, BRIAN MARK, PHD
PROFESSOR
DEPARTMENT OF APPLIED HEALTH SCIENCE
CENTER FOR SEXUAL HEALTH PROMOTION
SCHOOL OF PUBLIC HEALTH
INDIANA UNIVERSITY
BLOOMINGTON, IN 47405

SCIENTIFIC REVIEW OFFICER

RUBERT, MARK P, PHD
SCIENTIFIC REVIEW OFFICER
CENTER FOR SCIENTIFIC REVIEW
NATIONAL INSTITUTES OF HEALTH
BETHESDA, MD 20892

EXTRAMURAL SUPPORT ASSISTANT

STROTHERS, DIARA
EXTRAMURAL SUPPORT ASSISTANT
CENTER FOR SCIENTIFIC REVIEW
NATIONAL INSTITUTES OF HEALTH
BETHESDA, MD 20892

* Temporary Member. For grant applications, temporary members may participate in the entire meeting or may review only selected applications as needed.

Consultants are required to absent themselves from the room during the review of any application if their presence would constitute or appear to constitute a conflict of interest.
